# Congenital Neutropenia with Specific Granulocyte Deficiency Caused by Novel Double Heterozygous *SMARCD2* Mutations

**DOI:** 10.3390/hematolrep14030038

**Published:** 2022-09-09

**Authors:** Abukhiran Ibrahim, Anjali Sharathkumar, Heather McLaughlin, David Claassen, Sharathkumar Bhagavathi

**Affiliations:** 1 Department of Pathology, University of Iowa Carver School of Medicine, Iowa City, IA 52242, USA; 2 Stead Family Department of Pediatrics, University of Iowa Carver School of Medicine, Iowa City, IA 52242, USA; 3 Invitae Corporation, San Francisco, CA 94103, USA

**Keywords:** severe congenital neutropenia, *SMARCD2*, TPO-receptor agonist, Romiplostim

## Abstract

*SMARCD2* (SWI/SNF-related, matrix-associated, actin-dependent regulator of chromatin, subfamily D, member 2) is critical for myelopoiesis. Recently, bi-allelic *SMARCD2* mutations have been reported in five children, causing autosomal recessive congenital neutropenia with specific granulocytes deficiency (CN-SGD); a syndrome resulting in G-CSF resistant neutropenia, recurrent infections, and dysplastic myelopoiesis. We report a new case with CN-SGD caused by two novel heterozygous pathogenic variants in the *SMARCD2* gene (c.1081del (p.Gln361Argfs*15)), and (c.217C>T (p.Arg73*)). Treatment with the weekly dosing of thrombopoietin receptor agonist, Romiplostim, along with daily G-CSF transformed her clinical course, implying potential synergism. This report advances the understanding of CN-SGD caused by *SMARCD2* mutations.

## 1. Introduction

The *SMARCD2* (SWI/SNF-related, matrix-associated, actin-dependent regulator of chromatin, subfamily D, member 2) gene plays an important in role chromatin remodeling and myeloid differentiation in humans, zebrafish, and mice [1]. Recently, homozygous *SMARCD2* mutations were reported to cause autosomal recessive congenital neutropenia with specific granulocyte deficiency (CN-SGD) [1,2,3,4], a syndrome characterized by dysplastic myelopoiesis and sub-optimal phagocytotic activity, leading to recurrent skin and deep-seated pyogenic infections. These children present with life-threatening infections and granulocyte colony stimulating factor (G-CSF)-resistant neutropenia, requiring treatment with hematopoietic stem cell transplantation (HSCT). Other features include facial and skeletal dysmorphism, such as misaligned teeth and brittle nails, in addition to developmental delay and learning difficulties [1,2].

To date, five patients with CN-SGD have been reported, and understanding concerning the genotype-phenotype relationship is still evolving [1,2,3,4,5]. In this report, we elaborate the clinical course and management of a pediatric patient with CN-SGD caused by novel double heterozygous mutations within *SMARCD2*. This is the sixth case with CN-SGD caused by *SMARCD2* mutations.

## 2. Case Presentation

A 12-year-old African American girl was diagnosed with severe congenital neutropenia (SCN) at the age of 2 years. She had presented with recurrent infections since early infancy, absolute neutrophil count (ANC) < 0.5 103/mm^3^, and promyelocyte arrest in bone marrow. She had chronic mucopurulent rhinosinusitis, recurrent/chronic otitis media, and an extensive history of multiple bouts of superficial skin abscesses and deep cellulitis, requiring multiple hospitalizations. She had bilateral cervical and submandibular lymphadenopathy, gingival hypertrophy, and chronic stomatitis “cauliflower ear” deformity. Her growth and development were normal for her chronological age. Genetic testing, including normal karyotype, myelodysplastic syndrome (MDS) panel, elastase-2 gene, and HAX-1 gene mutations, was negative. Her immunoglobulin levels and lymphocyte subset analyses were normal. She was commenced on G-CSF, which required a dose increment to 20 microgram/kg/day due to apparent G-CSF resistance. Multiple blood smears showed Pelger–Huet anomaly, monocytosis, and intermittent circulating blasts. She underwent several surveillance bone marrow evaluations, cytogenetics, and fluorescence in situ hybridization (FISH) studies to rule out MDS/leukemia. Bone marrow evaluations since the age of 6 years showed dysmegakaryopoiesis, along with promyelocyte arrest and mild increase in reticulin fibrosis.

At the age of 11 years, she had sixteen hospitalizations with pseudomonas sepsis, Escherichia coli sepsis, candida infection, and rotavirus gastroenteritis. She had sacral abscess, multiple vaginal and perianal ulcerations, rectal abscess, and upper and lower GI bleeding, requiring multiple red cell transfusions. One of her hospitalizations was complicated by acute kidney injury and hypertension. Renal biopsy confirmed diagnosis of post-infectious glomerulonephritis, potentially from a skin infection, which was treated with steroids.

**Figure 1:**
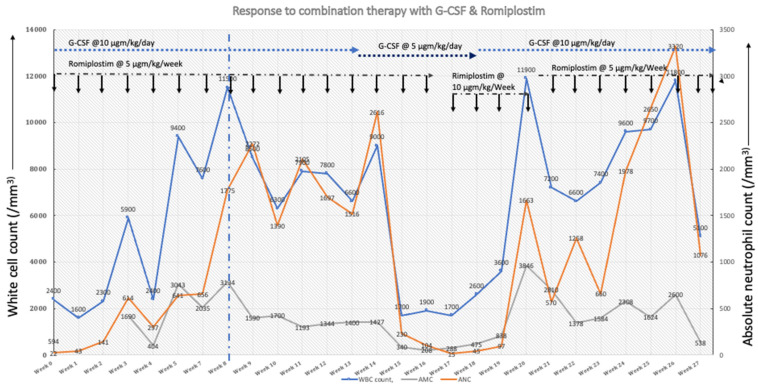
White cell, neutrophil, and monocyte response addition of Romiplostim during first 6 months of therapy. Reduction in dosage of G-CSF reduced neutrophil response during weeks 17 through 19.

**Figure 2:**
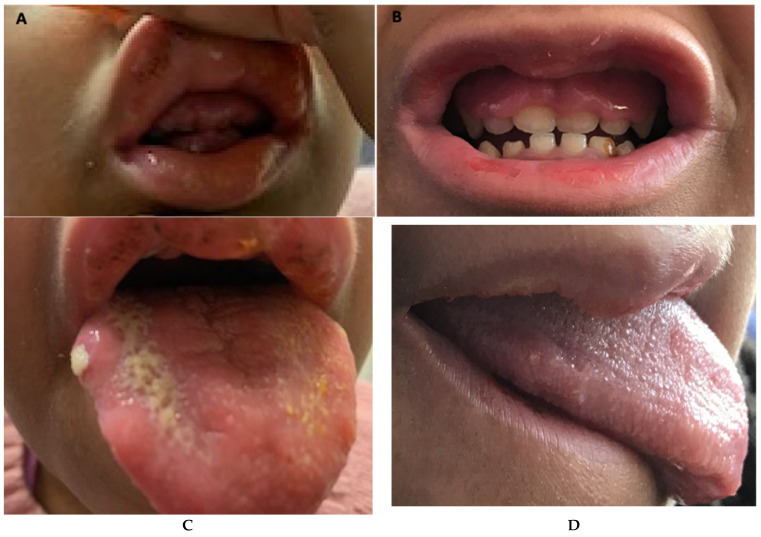
Clinical photographs showing improvement in gingival hypertrophy after addition of Romiplostim: (**A**) Gingival hypertrophy covering teeth before commencement of Romiplostim; (**B**) Improvement in gingival hypertrophy after 12 weeks of therapy with Romiplostim and G-CSF; (**C**) Stomatitis before therapy; (**D**) Resolution of stomatitis 8 weeks after Romiplostim therapy.

Because she had suboptimal and inconsistent response to G-CSF treatment throughout this period, the option of haploidentical HSCT from her relative was considered as she lacked a full-matched donor. To improve her ANC, the thrombopoietin receptor agonist (TPO-RA) Romiplostim was started at 5 microgram/kg/week due to its promising action on HSCs in patients with severe aplastic anemia (SAA) [6,7]. Romiplostim was chosen due to its parenteral mode of administration, ensuring absorption. Upon the addition of Romiplostim along with G-CSF (10 mcg/kg/day), her ANC recovered in 8 weeks (Figure 1), with improvement in her gingival hypertrophy and oral lesions (Figure 2). An attempt to reduce her G-CSF dose to 5 mcg/kg/day quickly dropped her ANC, requiring an increment in dosing. She remained on this combination regimen with G-CSF at 10 mcg/kg/day and Romiplostim at 5 mcg/kg/week for 6 months. Her bone marrow showed mild to moderate reticulin fibrosis. She relocated, and Romiplostim was discontinued. She underwent myeloablative haplo-identical HSCT from her half-sibling 3 months after discontinuation of Romiplostim. Pretransplant bone marrow testing showed reduction in her reticulin fibrosis. It is ~1.5 years since her HSCT, and she has had full engraftment.

Due to the complexity of her clinical course, clinical exome sequencing was performed using genomic DNA [8], which revealed two heterozygous pathogenic variants in the *SMARCD2* gene: NM_001098426.1 (c.1081del (p.Gln361Argfs*15) and c.217C>T (p.Arg73*)), explaining the etiology of her SCN. The phase of these variants is unknown because family member samples were not submitted for sequencing. However, they are expected to be in trans (e.g., on opposite chromosomes), explaining the etiology of her SCN.

Abbreviations: G-CSF, Granulocyte colony stimulating factor; WBC, white blood cell count; ANC, absolute neutrophil count; AMC, absolute monocyte count.

## 3. Discussion

This report shares the clinical course of a patient with CN-SGD caused by suspected compound heterozygous mutations within the *SMARCD2* gene. Although this is a sporadic case with an autosomal recessive inheritance, the clinical characteristics and bone marrow findings of our patient align with the previously described phenotype, except she had normal growth and neuro-development. Unlike previously reported cases, our patient developed clinical features, such as Bechet disease and post-infectious glomerulonephritis. Our patient showed evidence of early myelofibrosis at the age of 6 years, even before commencement of TPO-mimetic therapy. As expected, the myelofibrosis worsened after commencement of Romiplostim but reduced within 3 months after discontinuation of Romiplostim [9,10].

Table 1 summarizes the previous mutations within *SMARCD2* in five cases with CN-SGD. The two heterozygous novel variants that were identified in our case are considered pathogenic as both sequence changes create a premature translational stop signal in the gene that is predicted to result in an absent or disrupted protein product. Both variants are absent in the gnomAD population database (https://gnomad.broadinstitute.org/, accessed on 25 May 2022) and had not been reported in the literature in individuals with SMARCD2-related conditions.

The molecular mechanisms contributing to *SMARCD2*-related SCN and immune deficiency are well characterized [1,2,3]. SMARCD2-deficient bone marrow-derived CD34+ cells have impaired in vitro expansion and differentiation toward the neutrophilic lineage-causing SCN [3]. Furthermore, the SMARCD2-deficient neutrophils are shown to have sub-optimal phagocytic activity due to severely impaired chemotactic responses, which in turn causes abnormal disaggregation, locomotion, the defective in vitro-killing of Staphylococcus aureus, and lack lactoferrin, leading to susceptibility towards infection [2,4]. Interestingly, SMARCD2-deficient neutrophils show normal levels of myeloperoxidase and normal oxidative burst response. Furthermore, the phenotype of CN-SGD caused by *SMARCD2* [1] is similar to that caused by five-base pair deletion within the second exon of the *CEBPε* (CCAAT/enhancer binding protein epsilon) gene [11]. The *SMARCD2* gene product functions as a controller of early myeloid–erythroid progenitor cellular differentiation via interaction with transcription factor CEBPε [1]. As a result, mutations within these two genes lead to similar downstream effects and cause clinically similar phenotypes [12]. Unlike patients with CEBPε deficiency, most of the reported patients who had SMARCD2 deficiency showed evidence of myelodysplasia. Therefore, it is critical to identify the underlying molecular events to better characterize outcomes in children with CN-SGD. These differences can be attributed to the role of SMARCD2 in controlling early-stage differentiation of HSCs toward neutrophil granulocytes, while CEPBε controls terminal differentiation of neutrophils [13].

Our patient’s clinical course was altered after the addition of Romiplostim, TPO-RA. The rationale behind using Romiplostim in this desperate clinical setting deserves discussion. Thrombopoietin and TPO-RA induce hematopoietic stem and progenitor cells (HSPCs) through binding with the receptor c-mpl [14] and regulate hematopoiesis through its pleotropic actions [15,16]. These findings led to clinical trials evaluating TPO-RA as a therapeutic option for SAA with encouraging outcomes [6,7,16,17,18]. The dosing regimen in SAA was higher than immune thrombocytopenic purpura and ranged from 5 to 20 mcg/kg/dose [18,19]. We hypothesized that Romiplostim will offer therapeutic benefit through its synergistic action with G-CSF. Romiplostim, being an inducer of hematopoiesis, will induce HSPCs, consequently increasing the pool of common myeloid progenitors, while G-CSF, a myeloid lineage-specific chemokine, will induce the proliferation, differentiation, and maturation of myeloid/neutrophil precursors into mature neutrophils and release them into the bloodstream [20]. An attempt to reduce G-CSF dose immediately dropped our patient’s ANC, which indirectly provides evidence that Romiplostim helped induce the proliferation of HSPCs, yet a higher dose of G-CSF was needed for the subsequent maturation of granulopoiesis. While we do not have mechanistic data to support our hypothesis, it is possible that Romiplostim may offer a synergistic therapeutic benefit to children with SCN/CN-SGD who are resistant to G-CSF. Clearly, caution needs to be applied prior to using combination therapy as it may increase the risk of progression towards MDS/leukemia. Furthermore, our report is no exception to the inherent limitations associated with case studies, such as lack of ability to generalize, danger of over-interpretation, reporting bias, retrospective design, and inability to establish a cause-and-effect relationship. In conclusion, we share the clinical course of a patient with CN-SGD caused by novel heterozygous mutations within SMRCD2 and the short-term benefit of combination therapy with G-CSF and Romiplostim.

## Figures and Tables

**Table 1:** Review of the literature regarding severe congenital neutropenia caused by *SMARCD2* mutations. The two siblings and the first mutation, which produced three aberrant transcripts. All mutations resulted in frameshifts and premature sequence termination.

Author	Case Number	Molecular Mutations within *SMARCD2* Gene		
		Nucleotide Change	Protein Change	Location
van der Schim et al. [2]	Case 1	c.1181+1G>A	Ile362Cysfs*3 Ser394Argfs*1 Ile362Valfs*85	Intron 9
Witzel et al. [1]	Case 2	c.401+2T>C	p.Arg73Valfs*8	Intron 2
Witzel et al. [1]	Case 3 and 4	c.414_438dup	p.Gln147Glufs*5	Exon 3
Yucels et al. [3]	Case 5	c. 93del	p.Ala32Argfs*80	Exon 1
Our case	Case 6	c.217C>T c.1081del	p.Arg73* p.Gln361Argfs*15	Exon 2 Exon 8

Abbreviations: SNS: single nucleotide substitution. The asterisk represents HGVS nomenclature for a premature termination codon. These must remain to accurately describe the mutations.

## Data Availability

Data will be available upon request.

## Abbreviations

**Abbreviation**	**Expansion**	**Abbreviation**	**Expansion**
**ANC**	Absolute neutrophil count	**HSPC**	Hematopoietic stem progenitor cells
**SAA**	Severe aplastic anemia	**MDS**	Myelodysplastic syndrome
**CN-SGD**	Congenital neutropenia with specific granulocyte deficiency	**SCN**	Severe congenital neutropenia
**G-CSF**	Granulocyte Colony Stimulating Factor	*SMARCD2*	SWI/SNF-related, matrix-associated, actin-dependent regulator of chromatin, subfamily D, member 2
**HSCT**	Hematopoietic stem cell transplant		

## References

[1] Witzel M., Petersheim D., Fan Y., Bahrami E., Racek T., Rohlfs M., Puchałka J., Mertes C., Gagneur J., Ziegenhain C. (2017). Chromatin-remodeling factor SMARCD2 regulates transcriptional networks controlling differentiation of neutrophil granulocytes. Nat. Genet..

[2] van der Schim L.I., Sprenkeler E.G.G., Tool A.T.J., Abinun M., Grainger A., Engelhardt K.R., van Houdt M., Janssen H., Kuijpers T.W., Hambleton S. (2021). Defective neutrophil development and specific granule deficiency caused by a homozygous splice-site mutation in SMARCD2. J. Allergy Clin. Immunol..

[3] Yucel E., Karakus I.S., Krolo A., Kiykim A., Heredia R.J., Tamay Z., Cipe F.E., Karakoc-Aydiner E., Ozen A., Karaman S. (2021). Novel Frameshift Autosomal Recessive Loss-of-Function Mutation in SMARCD2 Encoding a Chromatin Remodeling Factor Mediates Granulopoiesis. J. Clin. Immunol..

[4] Gombart A.F., Shiohara M., Kwok S.H., Agematsu K., Komiyama A., Koeffler H.P. (2001). Neutrophil-specific granule deficiency: Homozygous recessive inheritance of a frameshift mutation in the gene encoding transcription factor CCAAT/enhancer binding protein—Epsilon. Blood.

[5] Wilson B.G., Roberts C.W.M. (2011). SWI/SNF nucleosome remodellers and cancer. Nat. Rev. Cancer.

[6] Lee J.W., Lee S.-E., Jung C.W., Park S., Keta H., Park S.K., Kim J.-A., Oh I.-H., Jang J.H. (2019). Romiplostim in patients with refractory aplastic anaemia previously treated with immunosuppressive therapy: A dose-finding and long-term treatment phase 2 trial. Lancet Haematol..

[7] Townsley D.M., Scheinberg P., Winkler T., Desmond R., Dumitriu B., Rios O., Weinstein B., Valdez J., Lotter J., Feng X. (2017). Eltrombopag Added to Standard Immunosuppression for Aplastic Anemia. N. Engl. J. Med..

[8] Xue Y., Ankala A., Wilcox W.R., Hegde M.R. (2015). Solving the molecular diagnostic testing conundrum for Mendelian disorders in the era of next-generation sequencing: Single-gene, gene panel, or exome/genome sequencing. Genet. Med..

[9] Boiocchi L., Orazi A., Ghanima W., Arabadjief M., Bussel J.B., Geyer J.T. (2012). Thrombopoietin receptor agonist therapy in primary immune thrombocytopenia is associated with bone marrow hypercellularity and mild reticulin fibrosis but not other stromal abnormalities. Mod. Pathol..

[10] Ghanima W., Geyer J.T., Lee C.S., Boiocchi L., Imahiyerobo A.A., Orazi A., Bussel J.B. (2014). Bone marrow fibrosis in 66 patients with immune thrombocytopenia treated with thrombopoietin-receptor agonists: A single-center, long-term follow-up. Haematologica.

[11] Lekstrom-Himes J.A., Dorman S.E., Kopar P., Holland S.M., Gallin J.I. (1999). Neutrophil-specific granule deficiency results from a novel mutation with loss of function of the transcription factor CCAAT/enhancer binding protein epsilon. J. Exp. Med..

[12] Wada T., Akagi T., Muraoka M., Toma T., Kaji K., Agematsu K., Koeffler H.P., Yokota T., Yachie A. (2015). A Novel In-Frame Deletion in the Leucine Zipper Domain of C/EBPε Leads to Neutrophil-Specific Granule Deficiency. J. Immunol..

[13] Khanna-Gupta A., Sun H., Zibello T., Lee H.M., Dahl R., Boxer L.A., Berliner N. (2007). Growth factor independence-1 (Gfi-1) plays a role in mediating specific granule deficiency (SGD) in a patient lacking a gene-inactivating mutation in the C/EBPepsilon gene. Blood.

[14] Kimura S., Roberts A.W., Metcalf D., Alexander W.S. (1998). Hematopoietic stem cell deficiencies in mice lacking c-Mpl, the receptor for thrombopoietin. Proc. Natl. Acad. Sci. USA.

[15] Olnes M.J., Scheinberg P., Calvo K.R., Desmond R., Tang Y., Dumitriu B., Parikh A.R., Soto S., Biancotto A., Feng X. (2012). Eltrombopag and improved hematopoiesis in refractory aplastic anemia. N. Engl. J. Med..

[16] Townsley D.M., Desmond R., Dunbar C., Young N.S. (2013). Pathophysiology and management of thrombocytopenia in bone marrow failure: Possible clinical applications of TPO receptor agonists in aplastic anemia and myelodysplastic syndromes. Int. J. Hematol..

[17] Gill H., Wong R., Kwong Y.-L. (2017). From chronic immune thrombocytopenia to severe aplastic anemia: Recent insights into the evolution of eltrombopag. Ther. Adv. Hematol..

[18] Kantarjian H., Fenaux P., Sekeres M.A., Becker P.S., Boruchov A., Bowen D., Hellstrom-Lindberg E., Larson R.A., Lyons R.M., Muus P. (2010). Safety and efficacy of romiplostim in patients with lower-risk myelodysplastic syndrome and thrombocytopenia. J. Clin. Oncol. Off. J. Am. Soc. Clin. Oncol..

[19] Lee J.W., Jang J.H., Lee S.-E., Jung C.W., Park S., Oh I.-H. (2016). Efficacy and Safety of Romiplostim in Patients with Aplastic Anemia Refractory to Immunosuppressive Therapy: 1-Year Interim Analysis of Phase 2 Clinical Trial. Blood Cells Mol. Dis..

[20] Metcalf D. (1988). Colony stimulating factors and hemopoiesis. Ann. Acad. Med. Singap..

